# Microbiological Insights into the Stress-Alleviating Property of an Endophytic *Bacillus altitudinis* WR10 in Wheat under Low-Phosphorus and High-Salinity Stresses

**DOI:** 10.3390/microorganisms7110508

**Published:** 2019-10-29

**Authors:** Zonghao Yue, Yihao Shen, Yanjuan Chen, Anwen Liang, Cuiwei Chu, Can Chen, Zhongke Sun

**Affiliations:** 1 College of Life Sciences and Agronomy, Zhoukou Normal University, Zhoukou 466001, China; yzh@zknu.edu.cn (Z.Y.); 201607030008@zknu.edu.cn (Y.S.); 201607080056@zknu.edu.cn (A.L.); 20171071@zknu.edu.cn (C.C.); 201111031@zknu.edu.cn (C.C.); 2 Henan Key Laboratory of Plant Molecular Breeding and Bioreactor, Zhoukou 466001, China; 3 School of Mechanical and Electrical Engineering, Zhoukou Normal University, Zhoukou 466001, China; 20172033@zknu.edu.cn

**Keywords:** *Bacillus altitudinis*, wheat, endophyte, abiotic stress, plant growth-promoting bacteria

## Abstract

An indole–3–acetic acid producing *Bacillus altitudinis* WR10 was previously isolated from the root of wheat (*Triticum aestivum* L.). In this study, the strain WR10 was used for relieving abiotic stresses in wheat under low phosphorus and high saline in hydroponic co-culture models. Significantly, strain WR10 improved wheat seed relative germination rate under salinity stress (200/400 mM NaCl) and the root dry weight in wheat seedlings under phosphorus stress (10 μM KH_2_PO_3_) when insoluble phosphates are available. To provide insights into its abiotic stress-alleviating properties, the strain was characterized further. WR10 grows well under different culture conditions. Particularly, WR10 resists salt (12% NaCl) and hydrolyzes both inorganic and organic insoluble phosphates. WR10 uses many plant-derived substrates as sole carbon and energy sources. It produces catalase, amylase, phosphatase, phytase, reductase, and 1–aminocyclopropane–1–carboxylate (ACC) deaminase. In addition, WR10 possesses long peritrichous flagella, and its biofilm formation, as well as phytase production, is induced by abiotic stresses. Overall, the salinity-alleviating property of WR10 in wheat can be attributed to its inherent tolerance to NaCl, formation of biofilm, and production of enzymes, like catalase, amylase, and ACC deaminase. Meanwhile, *B. altitudinis* WR10 reduces low-phosphorus stress in wheat by production of phosphatases and phytases in the presence of insoluble phosphates.

## 1. Introduction

Among various abiotic stresses, salinity stress and phosphorus deficiency are two worldwide problems and restrict intensive agriculture [1,2]. As one of the major abiotic stresses, salinity affects crop production in arid and semiarid areas, which grow wheat as the major crop. Salt stress causes adverse physiological and biochemical changes in plant seeds, and sensitively delays or prevents seed germination and seedling growth [3]. Salt-induced toxicity on wheat is mainly oxidative stress and ion toxicity [4,5]. Inadequate available phosphorus is another threat to plants in many cropping environments, especially in withered, calcareous, and alkaline soils [6]. Low phosphorus resulted in significant decrease of root dry weight, root length density, and grain yield in field-grown wheat [7]. Different cultivars have evolved different mechanism to cope with phosphate deficiency; e.g., cv. KN92 changed the morphology of its roots, while cv. SJZ8 increased the physiological activities in its roots [8]. As a major food crop in the world, relieving salinity and low-phosphorus stresses in wheat is undoubtedly important for the society sustainable development.

Except plant innate mechanisms, many plant growth-promoting bacteria (PGPB) also evolved mechanisms for improving nutrient acquisition and stresses tolerance in plants [9,10]. Among those PGPB, the genus *Bacillus* (*B.*) is well studied due to its wide presence and attractive properties [11]. The genus contains already 379 species (including sub species) and spreads in various environments, such as soils, water, marine sediments, and plants (according to the list of prokaryotic names with standing in nomenclature, http://www.bacterio.net/bacillus.html, retrieved on 2019.07.30). In particular, many of them protect versatile plants from kinds of abiotic stresses. For example, *B. amyloliquefaciens* confers tolerance to various abiotic stresses in rice [12]. *B. aryabhattai* and *B. siamensis* alleviate heat and drought stresses in Chinese cabbage [13]. *B. aryabhattai* and *B. mesonae* improve tolerance to salinity stress in tomato [14].

Some PGPB have also been isolated from the internal tissues of plants, and were termed endophytes [15]. We once isolated a strain *B. altitudinis* WR10 from the root of wheat (*Triticum aestivum* L. cv. Zhoumai 26) in a previous study. The strain significantly reduces iron stress in wheat seedlings, and generates indole–3–acetic acid (IAA) [16]. However, the type strain of *B. altitudinis* had been isolated from high-altitude air samples and two strains of *B. altitudinis* possessing potential plant-growth promoting traits were isolated from wheat rhizosphere [17,18]. Thinking *B. altitudinis* WR10 has a different habitat from these strains, we hypothesize the strain may have different characteristics and those characteristics may provide important insights into its beneficial effects on wheat. Therefore, we first examined its potential for alleviating two kinds of abiotic stresses in wheat and then characterized the morphological, physiological, and biochemical features of this strain.

## 2. Materials and Methods

### 2.1. Reagents, Media, and Growth Conditions

All chemicals, including NaCl, crystal violet, tri-calcium phosphate, calcium phytate, 1–aminocyclopropane–1–carboxylate (ACC), were ordered at analytic purity if possible (Macklin Inc., Shanghai, China). The low-salt Luria-Bertani (LB) medium was used for culturing bacteria (composition: 10 g/L peptone, 5 g/L yeast extract, 5 g/L NaCl, 1.5% agar when used for solid culturing). For assays of biofilm formation in bacteria, a modified LB medium (LBGM) was used, which is composed of LB broth supplemented with 1% of glycerol and 200 μM MnSO_4_. The Hoagland’s complete nutrient solution (in full or in half-strength) was used for growing wheat or co-culture of bacteria and plant. *B. altitudinis* WR10 was normally grown in standard LB broth by agitating at 150 rpm under 30 °C, except indicated elsewhere. Wheat seeds were sterilized by 75% ethanol for 5 min and then rinsed three times by distilled water. Wheat seedlings were grown at room temperature (20–30 °C) in humid condition (60–80%) under dark or light for 12 h every day.

### 2.2. Germination Test of Wheat Seeds under Salinity Stress

A hydroponic co-culture model was used to evaluate the effect of *B. altitudinis* WR10 on wheat under salinity stress [19]. In brief, 10^6^ cfu/mL WR10 cells and 300 ethanol sterilized seeds of *T. aestivum* L cv. Zhoumai 26 were co-cultured in twelve sets of Petri dishes each containing 25 seeds, as described previously [20]. To test the effect of WR 10 on salinity stress to wheat, four levels of NaCl (0, 100, 200, and 400 mM in final concentration) were added into Hoagland’s complete nutrient solution. According to previous reports, the relative germination rate (RGR) of wheat seeds was calculated after one-week incubation from three repeated Petri dishes.

### 2.3. Growth Test of Wheat Seedlings Under Low-Phosphorus Stress

To test the effect of WR10 on wheat under low-phosphorus stress, another hydroponic co-culture model was used with minor adjustment [16]. Briefly, wheat (*T. aestivum* L cv. Zhoumai 26, Zhoukou Agriculture Academy, Zhoukou, China) seeds were germinated in the dark on moist filter paper at 30 °C. After germination, 120 well-germinated seedlings (three days after seeding) were transferred into half-strength Hoagland’s medium without phosphorus. One day later, these seedlings were allocated into eight containers, with 15 in each. KH_2_PO_3_ was added to a final concentration of 10 μM for low-phosphorus stress, and to 500 μM as normal control. Pre-cultured WR10 cells were added or absented during co-culture. Insoluble Ca_3_(PO_4_)_2_ as inorganic phosphorus resource (250 μM) and calcium phytate (Ca–phytate, 100 μM) as organic phosphorus resource were added, respectively, into different containers to reach a final concentration of 500μM phosphorus. Fresh medium was exchanged after 48 h incubation. As a confirmed indicator, the root dry weight (RDW) in all seedlings was measured after two-week incubation and used for evaluation of growth response. Meanwhile, photometric determination of available phosphorus in the supernatant after 48 h incubation was carried out using the phosphovanadomolybdate complex [21].

### 2.4. Growth Test of B. altitudinis WR10 under Different Conditions

To further correlate the growth-promoting properties, *B. altitudinis* WR10 was characterized by growth test under different conditions. Growth was monitored at different temperatures (10, 20, 30, 37, 40, 50, 55, or 60 °C) and NaCl concentrations (0, 0.5, 1.0, 2.0, 4.0, 6.0, 8.0, 10.0, 12.0, or 14.0%, *w*/*v*) in tubes containing 5 mL liquid LB broth. Tolerance to pH was evaluated between 2.0 and 11.0 with intervals of one unit by adding concentrated HCl or 2 M NaOH solutions into 5 mL LB broth. Growth was also tested on LB agar supplemented with either 0.05% Ca_3_(PO_4_)_2_ or Ca–phytate as substrate.

### 2.5. Biochemical Analysis of B. altitudinis WR10

Experiments using API 50CHB (Bio Mérieux, Lyon, France) and Gen Ⅲ MicroStation (Biolog, Hayward, CA, USA) were carried out to evaluate the physiological and biochemical characteristics, in accordance with the manufacturers’ protocols. All experiments were conducted in LB broth at 30 °C for 24 h.

### 2.6. Enzymatic Analysis of B. altitudinis WR10 and Potential Genes 

The enzyme profile was investigated using API ZYM and API 20NE (Bio Mérieux, Lyon, France) in broth according to product guidelines. Phenotypic tests of different enzymes production were conducted on agar plate with different substrates. Briefly, the production of catalase was piloted using 3% H_2_O_2_ as substrate. Hydrolyzation of starch was tested with 2% soluble starch. Production of phytases was tested with 0.05% calcium phytate on agar plates. Production of ACC deaminase was tested with 3.0 mM ACC as sole nitrogen source on DF minimal medium agar plate as described in Reference [22]. The presence of genes encoding different enzymes was retrieved by keyword searching of the reference genome of *B. altitudinis* strain GR–8 (Accession No. CP009108.1). Strain GR–8 was selected because it has 99.50% identity in a marker gene *gyrB* to *B. altitudinis* strain WR10 (Accession No. KY416927.1) and its complete genome were annotated in the NCBI database.

### 2.7. Morphological Analysis and Biofilm Formation of B. altitudinis WR10

Cell morphology was observed using a Tecnai G2 F20 S–TWIN transmission electron microscope (TEM, FEI Company, Hillsboro, OR, USA). The ability of biofilm formation was assayed by the classical crystal violet staining, as described elsewhere, with minor modification [23]. Briefly, *B. altitudinis* WR10 cells were first grown in 5 mL of LB broth to exponential phase. Then, 20 μL cultures were added into the 96-well clear polystyrene microplates (Cat. 3370, Corning, NY, USA) filled with 180 μL of LBGM broth, or LBGM supplemented with different concentrations of NaCl (100, 200, 400 mM). The LBGM, a LB based medium supplemented with 1% of glycerol and 200 μM MnSO_4_ was used in this study, as it promotes biofilm formation in *Bacillus* spp [24]. Plates were sealed with lid and incubated statically at 30 °C for 48 h. Biofilm was stained with 20 μL of a 0.1% (*w*/*v*) crystal violet solution. Non-adherent bacteria were removed by washing with phosphate buffered saline (PBS). At last, biofilms stained crystal violet was released by the addition of 200 μL of 100% ethanol and biofilm formation was quantified by measuring absorbance at 562 nm in plate reader (Multiskan FC, Thermo, Germany).

### 2.8. Phytase Activity Assays under Different Levels Phosphorus

*B. altitudinis* WR10 was fermented in 5 mL LB broth supplemented with different concentrations (0, 10, 100, 500 μM) of KH_2_PO_3_, and agitated for 24 h at 37 °C. Distilled high-pure water without any phosphorus was used for medium preparation. All samples used for phytase activity assays were prepared by collection of supernatants after centrifugation (5000RCF, 5 min) of overnight culture broth. Phytase assay was adapted from the Phytex method as described in Reference [25]. In brief, 100 μL culture supernatant was mixed with equal volume 10.8 mM sodium phytate and reacted for 15 min at 37 °C, and then stopped by 200 μL 15% tricholoroacetic acid. One phytase unit (FTU) was defined as enzyme that catalyzes the release of 1μmol inorganic phosphate per minute.

### 2.9. Data Analysis

All data were mean of three independent experiments with replicates (*n* = 25 in wheat seeds germination test, *n* = 15 in wheat seedlings growth test) and expressed as mean ± standard deviations (SD). Statistical analysis was performed using SPSS19.0 by one-way ANOVA with Bonferroni post-tests for multiple comparisons (significant with *p* < 0.001) when necessary.

## 3. Results

### 3.1. B. altitudinis WR10 Alleviates Abiotic Stresses under Either High Saline or Low Phosphorus

**Figure 1:**
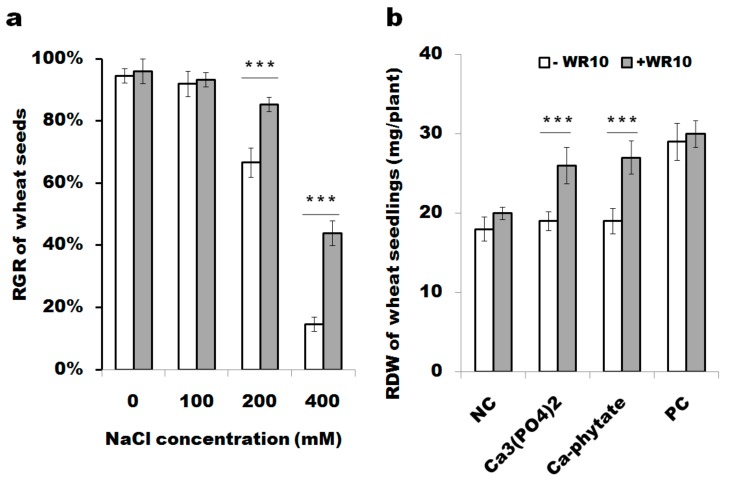
Test of *B. altitudinis* WR10 stress alleviating potential under either high saline or low phosphorus: (**a**), relative germination rate (RGR) of wheat seeds under high saline; (**b**), roots dry weight (RDW) of wheat seedlings under low phosphorus. The RGR of wheat seeds (*n* = 25) was calculated for evaluating salinity stress alleviating potential after culturing for one week under different concentration of NaCl (0, 100, 200, and 400 mM). Co-cultures were performed in Hoagland’s medium and the final cell density of WR10 was adjusted to 10^6^ cfu/mL. The RDW of wheat seedlings (*n* = 15) was recorded after two-week culture in a modified half-strength Hoagland’s medium without phosphorus resource, unless with extra addition of 10 μM KH_2_PO_3_ and other different phosphate resources (250 μM Ca_3_(PO_4_)_2_ or 100 μM calcium phytate). All roots from each plant were collected and oven dried before weighing RDW. Data are the mean of three independent experiments. Statistical analysis was performed using one-way ANOVA with Bonferroni post-test (*** *p* < 0.001).

As shown in Figure 1a, the RGR of wheat seeds was sharply decreased under 400 mM NaCl comparing to that under 0 mM NaCl, indicating severe stress of saline on wheat. Adding of *B. altitudinis* WR10 significantly improved RGR of wheat seeds under high salinity stress (increased from 20% to 60%, *p* < 0.001, *n* = 25). Meanwhile, adding of *B. altitudinis* WR10 does not influence seed RGR under 0 mM NaCl, and improve seed RGR under 200 mM NaCl. As shown in Figure 1b, low-phosphorus stress (10μM KH_2_PO_3_) led to a decrease of the RDW in wheat seedlings compared to those grown under normal conditions (e.g., 500μM KH_2_PO_3_), irrespective of the supplementation of WR10. In contrast, co-culture with WR10 reversed this adverse effect in the presence of either insoluble Ca_3_(PO_4_)_2_ or Ca–phytate. Significantly, the mean RDW increased from about 18 ± 1.5 mg to 26 ± 2.3 mg per plant after two weeks incubation (*p* < 0.001, *n* = 15).

### 3.2. B. altitudinis WR10 Resists High Saline and Grows under Different Conditions

**Figure 2:**
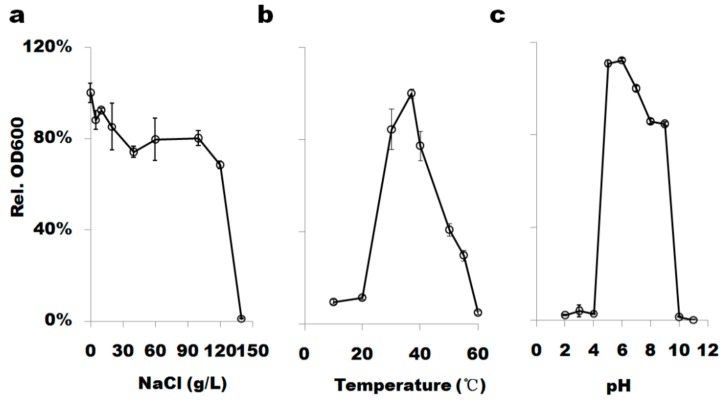
Growth of *B. altitudinis* WR10 under different conditions: (**a**), growth under different levels salt; (**b**). growth under different temperatures; (**c**), growth under different pH values. *B. altitudinis* WR10 was grown in Luria-Bertani based broth and incubated at 37 °C by agitating for 24 h. Data were the mean of three independent experiments.

As shown in Figure 2a, *B. altitudinis* WR10 grows within a wide range of sodium chloride. The tolerant range of NaCl is 0–12.0% (*w*/*v*) which means WR10 is extraordinarily resistant to salt as it grows well under as higher as 2 M NaCl. In addition, *B. altitudinis* WR10 grows under different temperature (ranges from 10 to 55 °C, with optimum at 30–40 °C, Figure 2b) and pH (ranges from 5.0 to 9.0, with optimum pH 5.0–6.0, Figure 2c).

### 3.3. B. altitudinis WR10 Forms Flagella and Produces Biofilm

**Figure 3:**
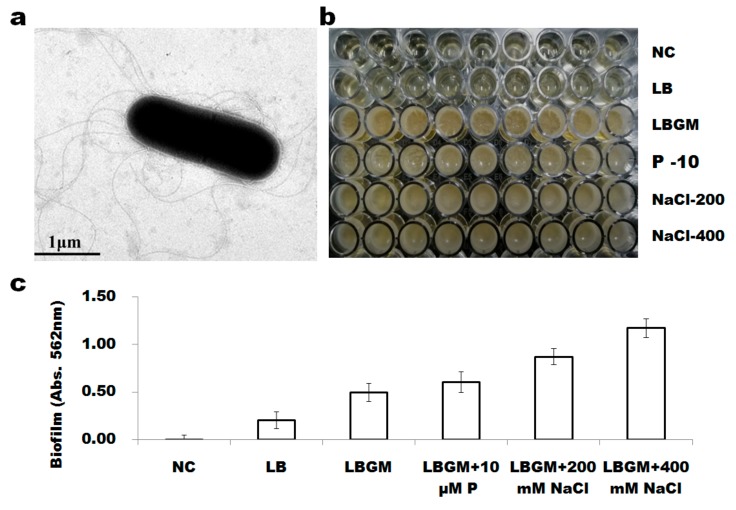
The morphology of *B. altitudinis* WR10 and its biofilm formation: (**a**). transmission electron microscope image of WR10; (**b**). phenotypes of WR10 biofilm formation in polystyrene microplates under static incubation grown in different media; (**c**). quantification of biofilms of WR10 by crystal violet staining. Data were mean of three independent experiments. NC, negative control, LB medium without inoculation of bacterium; LB, WR10 grown in Luria-Bertani medium (LB); LBGM, WR10 grown in LB supplemented with 1% of glycerol and 200 μM MnSO_4_; P–10, WR10 grown in LBGM + 10 μM KH_2_PO_3_; NaCl–200, WR10 grown in LBGM + 200 mM NaCl; NaCl–400, WR10 grown in LBGM + 400 mM NaCl.

The TEM imaging showed that strain WR10 was short, rod-shaped, and had many peritrichous flagella on the cell (Figure 3a). Statically growth experiment demonstrated *B. altitudinis* WR10 formed biofilm on the surface of polystyrene microplates in all media and much dense in LBGM broth than in LB broth (Figure 3b). Further quantification of biofilms by crystal violet staining revealed that both low phosphorus (P-10) and high salt (NaCl–200/400) increased WR10 biofilm formation, while salt had a much stronger influence on biofilm formation than phosphorus (Figure 3c). Besides this, high salt induced more biofilm than medium salt, and all were significant compared to that in LBGM broth (*p* < 0.001).

### 3.4. B. Altitudinis WR10 Ferments a Versatile of Plant-Derived Substrates

Results from API 50CHB test showed *B. altitudinis* WR10 ferments many carbon sources and some of them are enriched in plant, e.g., d–xylose, d–sorbitol, d–cellobiose, amygdalin, esculin, and salicin (see details in Supplementary information: Table S1). Inositol, a hydration product of phytate, can also be fermented by *B. altitudinis* WR10. Also, WR10 uses ferric citrate as carbon source to produce acid. Comprehensive results from the Biolog Gen III system with 95 carbon sources suggest WR10 uses various substrates as sole carbon and energy sources (Table S2). Substrates can be fermented by *B. altitudinis* WR10 in both API and Biolog Gen III tests were summarized (Table 1).

### 3.5. B. altitudinis WR10 Produces Many Stress-Alleviating Enzymes

According to the API ZYM test, *B. altitudinis* WR10 is positive for various enzymes, including alkaline phosphatase, acid phosphatase, and esterase (Table S3). In the catalase assay, gas bubble was observed. In the amylase and phytase assay, clear degradation halo was obvious around inoculated WR10 colonies. In the ACC deaminase assay, WR10 grows well on DF minimal agar using ACC as the sole nitrogen resource. WR10 can also reduce nitrate to nitrite (Table S4). In addition, many genes encoding these enzymes are presented in the genome of the reference strain *B. altitudinis* GR–8. For simplicity, we summarized these enzymes that may be important for the growth-promoting property of WR10, especially on alleviating abiotic stresses (Table 2).

### 3.6. B. altitudinis WR10 Degrades Insoluble Phosphate and Produces Phytases 

**Figure 4:**
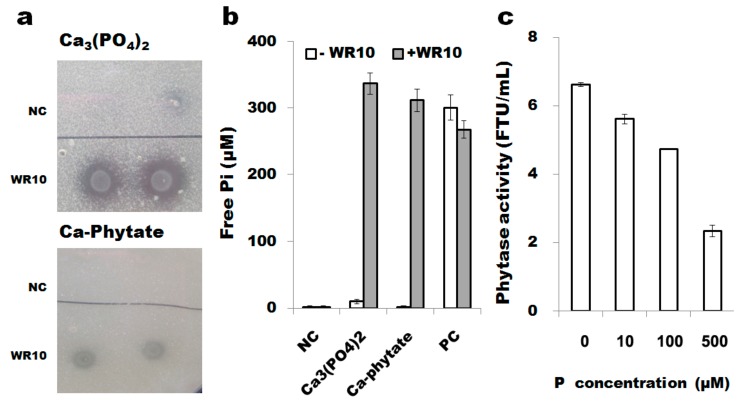
Insoluble phosphate degradation and phytases production by *B. altitudinis* WR10: (**a**), phenotypic degradation on agar plate; (**b**), free phosphorus in medium under different treatments; (**c**). phytases activity in the medium under different levels of P. For phenotypic assays, two microliters of pre-cultured *B. altitudinis* WR10 was spotted on LB agar supplemented with insoluble Ca_3_(PO_4_)_2_ or Ca–phytate and incubated at 37 °C for 24 h. Culture medium was used for assay of free phosphorus after centrifugation. For phytase enzyme activity assays, *B. altitudinis* WR10 was grown in LB broth supplemented with different concentrations of KH_2_PO_3_ (0, 10, 100, 500 μM) and incubated at 37 °C by agitating for 24 h. Supernatants were collected for enzymatic assay using the Phytex method. Data were mean of three independent experiments.)

Agar test with either 0.05% Ca_3_(PO_4_)_2_ or Ca–phytate as P sources suggests that *B. altitudinis* WR10 degrades them, as clear halo zone observable around seeded colonies (Figure 4a). Meanwhile, assay of plant available free phosphorus in the supernatant of medium suggests insoluble phosphate was effectively dissolved after adding WR10, as more than 300 μM free phosphorus can be detected in medium inoculated with bacteria after 48 h (Figure 4b). The production of phytases in *B. altitudinis* WR10 was further quantified by enzymatic activity assay. Phytase activity decreases with the increase of free phosphorus, suggesting the enzyme production may be induced by phosphorus deficiency and is inhibited by free phosphorus (Figure 4c).

## 4. Discussion

Due to significant yield-reducing effect in wheat, salinity stress is important. As a simple and sensitive phenotype, seed germination is adversely affected by salinity stress in many cereal plants [26]. Therefore, the RGR was calculated for assessing salinity tolerance [27]. The RGR of two wheat varieties decreased along with the increase of NaCl, though great variation in salt tolerance was observed in different varieties [28]. For *T. aestivum* L Zhoumai 26, it is relative resistant to salinity stress. As shown in Figure 1a, the RGR index is not affected under low salinity stress (100 mM NaCl) and is significantly reduced under medium and high salinity stresses (blank bars, 200 and 400 mM NaCl).

It is widely recognized that inoculation of PGPB results in increases in the growth and yield of different plants including wheat under salinity stress [29]. Particularly, PGPB could have a favorable effect on seed germination, such as increased RGR [30]. Inoculation of *Azotobacter* increased RGR of wheat seeds in soils, and the RGR can be even increased from about 30% to 80% under 300 mM NaCl after 11 days incubation [28,31]. In this study, adding of *B. altitudinis* WR10 significantly improved RGR of wheat seeds under high salinity stress (400 mM NaCl), increasing by approximately 30% (from roughly 15% to 44%) after 7 days of incubation. Considering moderate salinity stress is more common, we also tested the effect of WR10 on the RGR of other wheat cultivars under 200 mM NaCl. Our results showed constant improved RGR of other cultivars that are differently sensitive to salinity stress, after supplementation of WR10 (Table S5). The data demonstrated that the salinity stress-alleviating property of WR 10 is not cultivar dependent. However, more data on plant growth, like lengths of root and sprout, plant dry weight, and yield is not available at present, as there is no obvious growth under such condition within a week. 

The effect of PGPB on seed germination could be partially affected by hormones, including auxin that may be produced or regulated by bacteria [32]. It was revealed that IAA delays seed germination in wheat, and its exogenous application suppresses seed germination under high salinity in *Arabidopsis* [33,34]. However, a recent study showed that IAA levels are negatively related to the adverse effect of NaCl stress on wheat, suggesting that PGPB-produced IAA may be helpful for alleviating stress [35]. Many PGPB produces IAA, but the real effect of IAA on wheat seed germination under salinity stress is still unconfirmed. From this study, it seems *B. altitudins* WR10 produced IAA had no adverse effect on seed germination within 7 days, as there is no obvious difference of RGR under 0 mM NaCl.

Growth test showed *B. altitudinis* WR10 is extraordinarily resistant to NaCl, e.g., 12% (~2 M, Figure 2a), suggesting the strain is a halotolerant bacterium. Halotolerant bacteria grown in the range of 2–11% NaCl had been widely explored for promoting growth and augmenting tolerance to salinity in wheat [36,37,38]. They counter salinity stress by producing osmolytes that help plant keep normal osmosis and electrical conductivity [39]. Another beneficial characteristic of *B. altitudinis* WR10 is the formation of biofilm and its increase, along with the concentration of NaCl (Figure 3b,c). Bacterial formation of biofilm indirectly contributes to interacted plant salt tolerance [40,41]. Biofilm enhances bacteria surface attachment in roots, preserves proper moisture, and protects roots from drought stress [42]. Biofilm on root surface also restricts the Na^+^ importation into roots, as exopolysaccharides (EPS) in biofilm react with dissociated Na^+^ ions and chelate them [43,44]. Therefore, biofilm formation should be another contributor to the salt stress alleviating effect of *B. altitudinis* WR10.

As determined, *B. altitudinis* WR10 produces versatile enzymes, including catalase, amylases, and ACC deaminase though the corresponding genes of some of them were not annotated on the reference genome (Table 2). However, potential genes for those unannotated can be found using other analytic methods (e.g., conserved domain search and relatedness search). For example, a hypothetical protein encoded by gene ID12_14475 has an alpha amylase catalytic domain (cl07893). Two genes (ID12_01700 and ID12_03480) annotated as pyridoxal–phosphate dependent aminotransferases have a domain (cl00342) that corresponding to the ACC deaminase. As an antioxidant enzyme, catalase converts H_2_O_2_ to water and keeps reactive oxygen species (ROS) level down [45]. So, catalase-producing WR10 may protect wheat cells from stress-stimulated ROS induced apoptosis. Previous studies proved the starch granule deposition and hydrolase activity in cereal grains are important to seed germination, and thus amylase is also a key determinant [46]. Besides this, two halotolerant PGPB enhanced tolerance to salinity in wheat because of production of ACC deaminase and antioxidant enzymes [47]. A recent study also displayed that significantly declined stress stimulated ethylene levels (~60%) is virtually associated with bacterial ACC deaminase activity [48]. Higher RGR of wheat seeds was also observed in this study when inoculated with the ACC deaminase–producing *B. altitudinis* WR10 under salinity stress. The result might because of degradation of ACC, a precursor for ethylene production in plants, which is increased under salinity stress.

At last, lack of plant available phosphorus is common, especially in withered, calcareous, and alkaline soils. The adverse effect of phosphorus deficiency is even more severe on plants than salinity stress and its impact is pre-dominant when they were concomitantly exposed [49]. Low phosphorus hampers plant growth, e.g., decreasing weights of root and shoot in plant seedlings. Co-culture with *B. altitudinis* WR10 reversed this phenotype in the presence of either Ca_3_(PO_4_)_2_ and Ca–phytate (Figure 1b). The result is same to several previous studies that improved wheat available phosphorus and augmented phosphorus uptake after inoculation of different PGPB [50,51,52]. As a common feature of phosphate-solubilizing bacteria, they can degrade insoluble phosphates (either the inorganic or the organic or both of them) as demonstrated in Figure 4a. Co-culture assay suggests that insoluble phosphate was effectively dissolved after adding WR10, as high levels free phosphorus can be detected in the supernatants of medium (Figure 4b). Phytase specifically degrades phytate, a main form of organic phosphate. Although no gene in *B. altitudinis* with high domain (cl17685) identity to *B. subtilis* phytase, there was one gene (ID12_12285) was annotated as inositol monophosphatase, which degrades the intermediate of phytate. Therefore, phosphatases and phytases responded for the low-phosphorus stress alleviating effect of *B. altitudinis* WR10 on wheat. Nevertheless, to confirm those insights revealed in the study are major contributors to the stress-alleviating properties of *B. alitudinis* WR10, comparison with mutants are necessary. To explore the potential of WR10, further filed study using different cultivars in different regions is needed, and data on grain yield is definitely vital. 

## 5. Conclusions

Collectively, the study showed *B. altitudinis* WR10 is a halotolerant endophyte that protects wheat from abiotic stresses under either high saline or low phosphorus. Considering that WR10 forms flagella and ferments many plant-derived substrates, the strain may have evolved a close symbiotic relationship with wheat. The stress-alleviating properties as determined in this study can be explained from a microbiological aspect. That is, WR10 relieves salinity stress through secreting osmolytes, producing catalase, amylase, and ACC deaminase, as well as forming biofilm; and WR10 alleviates low-phosphorus stress by phosphatases and phytases degradation of insoluble phosphate.

## Figures and Tables

**Table 1:** A consensus profile of substrates fermented by *B. altitudinis* WR10.

Substrates	Results
Control	−
d–glucose	++
d–fructose	++
d–mannitol	++
d–cellobiose	++
d–maltose	++
d–melibiose	++
d–trehalose	++
d–raffinose	+
d–turanose	+
Methyl–d–glucopyranoside	+
Inositol	+

++, strongly positive; +, weakly positive; −, negative.

**Table 2:** Potential stress-alleviating enzymes produced by *B. altitudinis* WR10.

Enzymes	Phenotypes	Potential Function	Reference Gene(s) Tag
catalase	+	converts hydrogen peroxide to water and keeps ROS level down	ID12_04890, ID12_09825
amylase	+	hydrolyzes starch	not available
Alkaline phosphatase	+	hydrolyzes phosphate	ID12_09845, ID12_18380, ID12_18885
(acid) phosphatase	+	hydrolyzes phosphate	ID12_02705, D12_10250, ID12_10605, D12_11490, ID12_13905, D12_13990, ID12_14990
phytase	+	hydrolyzes phytate	not available
1–aminocyclopropane–1–carboxylate (ACC) deaminase	+	declines stress induced ethylene	not available
nitroreductase	+	defenses against oxidative stress	ID12_02145, D12_04220, ID12_07940, D12_09215, ID12_14945
nitrite reductase	+	reduces nitrite to NO, alleviates oxidative stress	ID12_14600, D12_14605, ID12_14610, ID12_14615

## Supplementary Materials

Supplementary materials can be found at https://www.mdpi.com/2076-2607/7/11/508/s1.
