# Supplementary material for: Microbiological Insights into the Stress-Alleviating Property of an Endophytic Bacillus altitudinis WR10 in Wheat under Low-Phosphorus and High-Salinity Stresses
Source: Microorganisms. 2019 Oct 29;7(11):508. doi: 10.3390/microorganisms7110508 (PMC6920878; doi:10.3390/microorganisms7110508)
Supplement: Supplementary file 1 [file microorganisms-07-00508-s001.pdf]

Supplementary information

Table S1 Physiological characteristics of *Bacillus altitudinis* WR10 evaluated by API 50CHB

**Substrates**	**Results**
Control	-
Glycerol	++
Erythritol	-
D-arabinose	-
L-arabinose	+
D-ribose	+
D-xylose	+
L-xylose	-
D-adonitol	-
β-methyl-D-xylopyranoside	-
D-galactose	+
D-glucose	++
D-fructose	++
D-mannose	++
L-sorbose	-
L-rhamnose	+
dulcitol	-
inositol	+
D-mannitol	+
D-sorbitol	+
α-mehyl-D-mannopyranoside	-
α-mehyl-D-glucopyranoside	+
N-acetyl-D-glucosamine	+
Amygdalin	+
Arbutin	++
Esculin ferric citrate	++
Salicin	++
D-cellobiose	++
D-maltose	++
D-lactose	-
D-melibiose	+
Sucrose	++
D-trehalose	++
Inulin	-
D-melezitose	-
D-raffinose	+
Starch	+
Glycogen	+
Xylitol	-
Gentiobiose	-
D-turanose	+
D-lyxose	-

D-tagatose	-
D-fucose	-
L-fucose	-
D-arabitol	-
L-arabitol	-
Potassium gluconate	+
Potassium 2-ketogluconate	-
Potassium 5-ketogluconate	-

++, strongly positive; +, weakly positive; -, negative

Table S2 Biochemical characteristics of *Bacillus altitudinis* WR10 evaluated by BiologGen III

Carbon sources	Results
Negative Control	-
Positive Control	++
Dextrin	-
D-maltose	++
D-trehalose	++
D-cellobiose	++
Gentiobiose	-
Sucrose	-
D-turanose	++
Stachyose	-
D-raffinose	-
α-D-lactose	-
D-melibiose	-
β-methyl-D-glucoside	++
D-salicin	-
N-acetyl-D-glucosamine	-
N-acetyl-β-D-mannosamine	-
N-acetyl-D-galactosamine	-
N-acetyl neuraminic acid	-
D-glucose	++
D-mannose	-
D-fructose	++
D-galactose	-
3-methyl glucose	-
D-fucose	-
L-fucose	-
L-rhamnose	-
Inosine	-
1% Sodium lactate	-
Fusidicacid	-
D-serine	-
D-sorbitol	-
D-mannitol	++
D-arabitol	++
myo-Inositol	++
Glycerol	-
D-glucose-6-phosphate	-
D-fructose-6-phosphate	-
D-aspartic acid	-
D-serine	-

Troleandomycin	-
Rifamycin SV	-
Minocycline	++
Gelatin	++
Glycyl-L-proline	-
L-alanine	++
L-arginine	-
L-aspartic acid	-
L-glutamic acid	-
L-histidine	++
L-pyroglutamic acid	-
L-serine	-
Lincomycin	-
Guanidine HCl	-
Niaproof 4	-
Pectin	++
D-galacturonic acid	-
L-galactonicacid lactone	-
D-gluconic acid	-
D-glucuronic acid	-
Glucuronamide	-
Mucicacid	-
Quinicacid	-
D-saccharicacid	-
Vancomycin	-
Tetrazolium violet	++
Tetrazolium blue	-
p-hydroxy-phenylacetic acid	-
Methyl pyruvate	-
D-Lactic acid methyl ester	-
L-lactic acid	-
Citric acid	-
α-keto-glutaric acid	-
D-malic acid	-
L-malic acid	-
Bromo-succinic acid	-
Nalidixic acid	-
Lithium chloride	-
Potassium tellurite	-
Tween 40	-
γ-amino-butryricacid	-
α-hydroxy-butyric acid	++
β-hydroxy-D,L-butyric acid	-

α-keto-butyric acid	-
Acetoacetic acid	-
Propionic acid	-
Acetic acid	-
Formic acid	-
Aztreonam	-
Sodium butyrate	-
Sodium bromate	-

++, strongly positive; +, weakly positive; -, negative

Table S3. Enzymatic profile of *Bacillus altitudinis* WR10 evaluated by API ZYM.

**Determined enzymes**	**Results**
Control	-
Alkaline phosphatase	+
Esterase(C4)	+
Esterase lipase(C8)	+
Lipase(C14)	-
Leucine arylamidase	-
Valine arylamidase	-
Cystine arylamidase	-
Trypsin	-
α-chymotrypsin	-
Acid phosphatase	+
Naphthol-AS-BI-phosphohydrolase	-
α-galactosidase	-
β-galactosidase	-
β-glucuronidase	-
α-glucosidase	-
β-glucosidase	-
N-acetyl-β-glucosamimidase	-
α-mannosidase	-
α-fucosidase	-

+, positive; -, negative

Table S4 Biochemical characteristics of *Bacillus altitudinis* WR10 evaluated by API 20NE

Substrates	reaction/enzyme	Results
KNO_3	$NO_3 \rightarrow NO_2$	+
	$NO_3 \rightarrow N_2$	-
Tryptophane	Indole production	-
D-Glucose	Acidification	+
Arginine	Arginine dihydrolase	-
Urea	Urease	-
Esculin ferric citrate	Hydrolysis (β-glucosidase)	+
Gelatin	Hydrolysis (protease)	+
p-nitrophenyl-β-D-galactopyranoside	β-galactosidase	+
D-Glucose	Assimilation	-
L-arabinose	Assimilation	+
D-mannose	Assimilation	-
D-mannitol	Assimilation	-
N-acetyl-D-glucosamine	Assimilation	+
D-maltose	Assimilation	+
Gluconate	Assimilation	+
Capric acid	Assimilation	-
Adipic acid	Assimilation	-
Malic acid	Assimilation	-
Sodium citrate	Assimilation	+
Phenylacetic acid	Assimilation	-

+, positive; -, negative

Table S5 The relative germination rate of different wheat cultivars under 200mM NaCl

**Cultivar**	**NaCl sensitivity**	**WR10 -**	**WR10 +**
Zhoumai 26	Moderate resistant	68%	82%
Zhoumai 36	Highly sensitive	8%	26%
Xilong 1376	Highly resistant	83%	98%
Xilong 2611	Slight resistant	50%	75%

Note: WR10 -, without addition of *B. altitudinis* WR10; WR10 +, supplemented with 10^6cfu/mL *B. altitudinis* WR10 cells. Co-culture was conducted in Hoagland's complete nutrient solution for a week. Data were mean of 25 seeds in triplicate experiments.
